# Fluorogenic Tissue-Based Assessment of Acid Ceramidase Activity

**DOI:** 10.21769/BioProtoc.5778

**Published:** 2026-08-20

**Authors:** Arielle Manabat, Debora Russo, Ilaria Penna, Rita Scarpelli, Laura Volpicelli-Daley

**Affiliations:** 1 Killion Center for Neurodegeneration and Experimental Therapeutics, The University of Alabama at Birmingham, Birmingham, AL, USA; 2 Structural Biophysics Facility, Fondazione Istituto Italiano di Tecnologia, Genova, Italy; 3 Medicinal Chemistry and Technologies for Drug Discovery and Delivery Facility, Fondazione Istituto Italiano di Tecnologia, Genova, Italy

**Keywords:** ASAH1, Acid ceramidase, Ceramides, Sphingolipids, Neurodegeneration, Farber disease

## Abstract

Acid ceramidase (aCDase) is a lysosomal amidase that catalyzes the hydrolysis of sphingolipids (SphL), including ceramides and glucosylceramides. Altered expressions of aCDase are associated with several pathological conditions, such as cancer, inflammation, pain, and pulmonary disorders. aCDase activity is reduced in Farber disease, spinal muscular atrophy with progressive myoclonic epilepsy, diabetes, and cardiovascular disease. Recent reports suggest that aCDase inhibition may be an emerging strategy for treating several SphL-related neurodegenerative conditions, such as Krabbe, Gaucher, and Parkinson’s disease, due to its role in the accumulation of glycosphingolipids. Therefore, the development of a tissue-based aCDase activity assay has potential applications in clinical diagnostics and drug discovery, enabling the evaluation of the onset and progression of disease from biological samples of patients, drug-target engagement analysis, and identification of biomarkers. Here, we report a detailed protocol for detecting aCDase activity in tissue lysates, using Rbm14-12 as a specific fluorogenic substrate for aCDase. Assay protocol optimization, including a procedure for the preparation and storage of tissue lysates and the identification of optimal protein tissue lysate amounts and substrate concentrations based on kinetic enzymatic parameter analyses, is described.

Key features

• This protocol relies on the use of the Rbm14-12 fluorogenic substrate.

• This protocol was developed out of a need to measure the target engagement of an aCDase-targeting therapeutic in a Parkinson’s disease-related animal model.

• This protocol can be broadly useful for sensitively measuring aCDase activity in central and peripheral organ tissues.

## Graphical overview


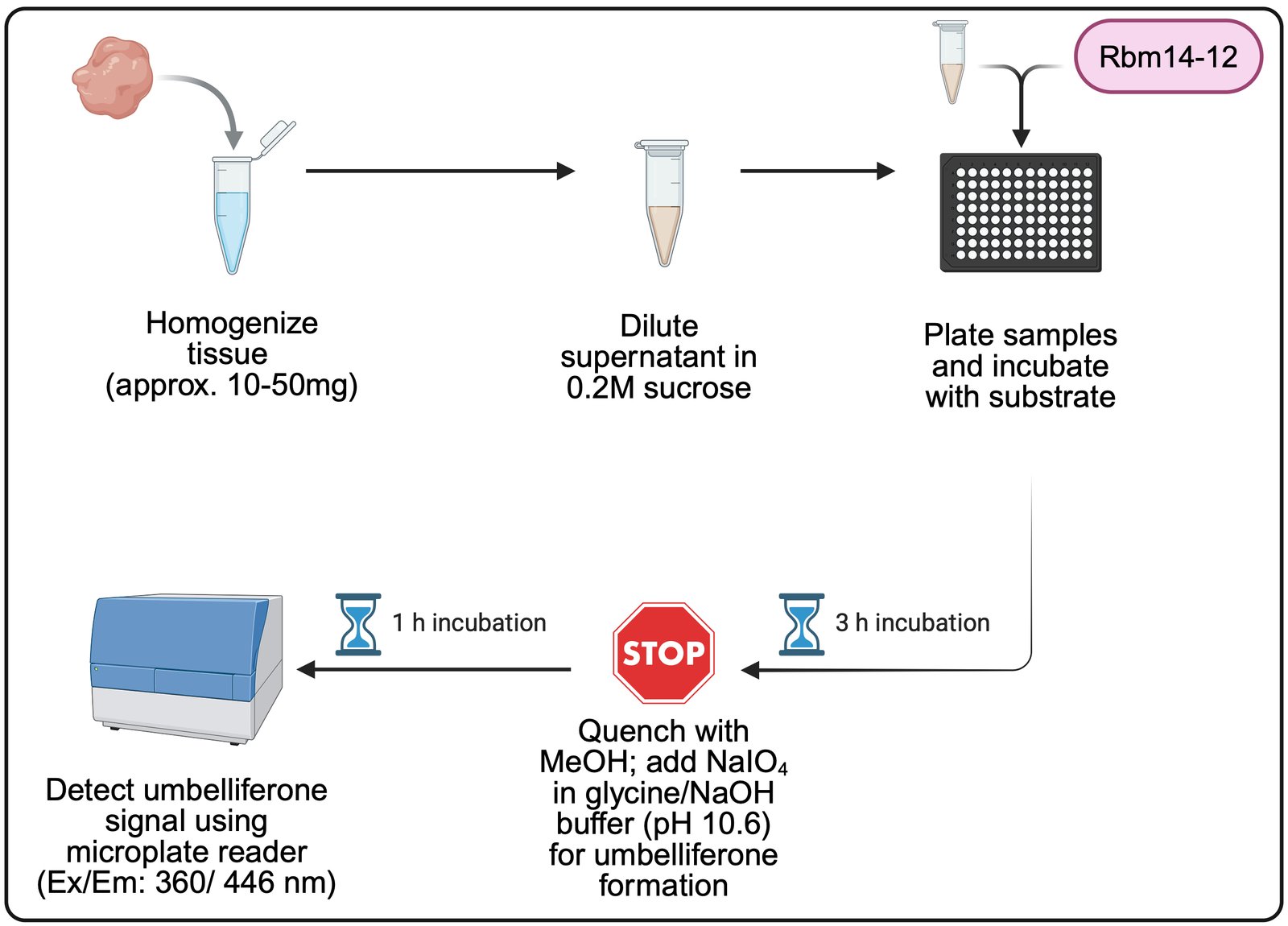


**Overview of the fluorogenic tissue-based assessment of acid ceramidase activity methodology.** MeOH, methanol; NaIO_4_, sodium periodate; NaOH, sodium hydroxide. NaIO_4_ solution is prepared in glycine/NaOH buffer (pH 10.6). Created with BioRender.com.

## Background

Acid ceramidase (aCDase, encoded by the *ASAH1* gene) is a cysteine amidase that hydrolyzes ceramides (Cer) to generate sphingosine and fatty acids. aCDase can also deacylate glucosylceramides (GlcCer) to form glucosylsphingosine (GlcSph) [1–5]. aCDase is localized within the lysosomal compartments and has a pH optimum of 4.5 to 5.0 [6–8]. Mutations in the *ASAH1* gene associate with lysosomal storage disorder, Farber disease (FD), and spinal muscular atrophy with progressive myoclonic epilepsy (SMA-PME) [9]. In the presence of mutant *ASAH1*, aCDase activity is reduced, leading to the buildup of lysosomal Cer in cells throughout the body [10].

Diagnosing FD based on assays that determine aCDase activity has been challenging, often requiring the use of radiolabeled substrates, specialized equipment, non-selectivity, and significant amounts of biological material, which is not always feasible [11,12]. A cell-based aCDase activity assay was previously developed using the fluorogenic Rbm14–12 substrate and a series of FD patient-derived cell lines, demonstrating its usefulness for diagnosing FD [13]. According to this previous protocol, aCDase hydrolyzes the amide bond of Rbm14-12 to form an aminodiol, which is oxidized by sodium periodate; the oxidized product (aldehyde) then undergoes β-elimination at basic pH, leading to the release of umbelliferone, a fluorescent coumarin-derived compound that can be measured with a microplate reader [13].

Evaluating aCDase activity has implications beyond FD and SMA-PME. In recent years, *ASAH1* expression and aCDase activity have been studied in the context of oncology, notably due to the overexpression of *ASAH1* in various tumor types [14–16]. aCDase activity has also been studied in the context of cardiovascular disease, as well as in aging, immunity, diabetes, and inflammation [17–20]. Most recently, aCDase has also been investigated for its role in some neurological disorders, such as Gaucher disease, Krabbe disease, Alzheimer’s disease, and Parkinson’s disease, due to its potential role in the accumulation of neurotoxic glycosphingolipids [21–24]. With the increasing relevance of aCDase in a variety of diseases, being able to accurately detect its activity in tissue lysates is fundamental. aCDase activity measurements are particularly valuable in preclinical validation studies in a variety of disease animal models to predict the clinical translation of targeting aCDase as a valuable therapeutic strategy.

Here, we describe a protocol for a tissue-based aCDase activity assay using the Rbm14-12 fluorogenic substrate. We optimized this protocol to measure aCDase activity in the brain of a Parkinson’s disease mouse model. This cost-effective, not laborious, and efficient aCDase activity assay has broad applicability for a variety of tissue types and animal models. The specific reagents needed for tissue homogenization, the optimal protein tissue lysate, and Rbm14-12 substrate concentrations were all determined in brain tissue lysates of GBA1^+/+^ wild-type (WT) mice. Key considerations for optimizing the assay for other tissue types are included.

## Materials and reagents

**Biological materials**

1. GBA1^+/+^ mouse brain tissue (WT mice, offspring of GBA1^+/L444P^ heterozygotes originally obtained from the University of North Carolina at Chapel Hill Mutant Mouse Resource and Research Center, catalog number/origin: MMRRC-000117)

**Reagents**

1. Dimethyl sulfoxide (DMSO) (Fisher Scientific, catalog number: D12345)

2. D-Sucrose (Fisher Scientific, catalog number: BP220-10)

3. EDTA (0.5 M, pH 8.0) (Fisher Scientific, catalog number: 15575020)

4. Glycine (Fisher Scientific, catalog number: BP381-500)

5. MeOH (Fisher Scientific, catalog number: A411-4)

6. Sodium periodate (NaIO_4_) (Millipore Sigma, catalog number: 311448-5G)

7. Pierce BCA Protein Assay kit (Fisher Scientific, catalog number: 23225)

8. Rbm14-12 substrate (Avanti Polar Lipids, catalog number: A86855)

9. Sodium acetate (1 M, pH 4.5) (Fisher Scientific, catalog number: J63669.AE)

10. Sodium chloride (NaCl) (Fisher Scientific, catalog number: BP358-212)

11. Sodium hydroxide (NaOH) (1 M) (Fisher Scientific, catalog number: SS266-1)

12. Tris base (Fisher Scientific, catalog number: BP152-500)

13. Umbelliferone (Millipore Sigma, catalog number: H24003)

**Solutions**

1. Assay solution (see Recipes)

2. Detergent-free lysis buffer (see Recipes)

3. 100 mM glycine/NaOH buffer (see Recipes)

4. 4 mM Rbm14-12 substrate stock solution (see Recipes)

5. 25 mM Sodium acetate buffer (see Recipes)

6. 2.5 mg/mL NaIO_4_ solution (see Recipes)

7. 0.2 M sucrose solution (see Recipes)

8. 5 mM umbelliferone stock solution (see Recipes)

**Recipes**

**1. Assay solution (per well)**

**Table:** 

Reagent	Final concentration	Volume
25 mM sodium acetate	18.6 mM	74.875 μL
Rbm14-12 substrate stock solution	5 μM*	0.125 μL
Fixed amount of protein	10 μg*	25 μL
Total	n/a	100 μL

*Conditions are to be optimized by the experimenter based on tissue type and animal model. See the aCDase activity assay reagent calculator in Supplementary information, Template 1, to easily calculate the amount of 4 mM Rbm14-12 substrate and the volume of 25 mM sodium acetate required to prepare the desired substrate concentration. For example, for a desired Rbm14-12 substrate concentration of 5 μM, 0.125 μL of 4 mM Rbm14-12 stock solution would be required per 100 μL of total volume. This can be scaled up using the aCDase activity assay reagent calculator in Template 1, so that a multi-channel pipette can be used to add 75 μL of assay solution to each necessary well.

The assay solution should be made fresh the day of the experiment.

**2. Detergent-free lysis buffer**

**Table:** 

Reagent	Final concentration	Volume
Tris, pH 8.0	50 mM	2.5 mL
5 M NaCl	150 mM	1.5 mL
0.5 M EDTA, pH 8.0	5 mM	0.5 mL
MilliQ water	n/a	45.5 mL
Total	n/a	50 mL

The detergent-free lysis buffer can be stored at room temperature (20–25 °C) for long-term storage. Collected protein supernatants using the detergent-free lysis buffer can be stored at -80 °C for long-term storage. Please note that protease and phosphatase inhibitors are omitted from the lysis buffer. To prevent any changes in enzyme stability, it is recommended to use chilled buffers, keep samples on ice, and work as quickly as possible while maintaining accuracy.

**3. 100 mM glycine/NaOH buffer**

**Table:** 

Reagent	Final concentration	Amount or volume
Glycine	n/a	1.875 g
MilliQ water	n/a	200 mL
1 M NaOH	n/a	to adjust pH to 10.6
MilliQ water	n/a	Bring to 250 mL
Total	100 mM	250 mL

The 100 mM glycine/NaOH buffer should be stored at 4 °C for up to two weeks, as long as the pH remains stable and unchanged. Please note that it is recommended to add 1.875 g of glycine to 200 mL of MilliQ water. Adjust the pH to 10.6 by slowly adding 1 M NaOH while stirring. After the solution reaches a pH of 10.6 based on the pH meter, add enough MilliQ water to bring the final volume to 250 mL. Mix thoroughly and verify the pH, adjusting if necessary.

**4. 4 mM Rbm14-12 substrate stock solution**

**Table:** 

Reagent	Final concentration	Amount or volume
Rbm14-12	4 mM	1 mg
DMSO	n/a	541.6 μL

Due to the volatility of ethanol (EtOH), it is recommended to dissolve Rbm14-12 in DMSO, rather than EtOH. The Rbm14-12 substrate stock solution in DMSO (4 mM) should be stored at -20 °C for <1 month in an amber glass vial wrapped in foil. It is recommended to aliquot the DMSO stock solutions of Rbm14-12 prior to storage to minimize freeze-thaw cycles.

**5. 25 mM sodium acetate buffer**

**Table:** 

Reagent	Final concentration	Amount or volume
1 M sodium acetate, pH 4.5	25 mM	1.25 mL
MilliQ water	n/a	48.75 mL
Total	25 mM	50 mL

The 25 mM sodium acetate buffer can either be stored at 4 °C or room temperature (20–25 °C) for up to two weeks, as long as the pH (4.5) remains stable and unchanged.

**6. 2.5 mg/mL NaIO_4 _solution**

**Table:** 

Reagent	Final concentration	Amount or volume
NaIO_4_	2.5 mg/mL	2.5 mg
100 mM glycine/NaOH buffer	1 mL	1 mL

The 2.5 mg/mL NaIO_4_ solution should be made fresh the day of the experiment.

**7. 0.2 M sucrose solution**

**Table:** 

Reagent	Final concentration	Volume
1 M sucrose	0.2 M	12.5 mL
MilliQ water	n/a	50 mL
Total	n/a	62.5 mL

The 0.2 M sucrose solution can be stored at 4 °C for up to two weeks, as long as the pH (6.0–6.5) remains stable and unchanged. Please note: to prepare the 1 M sucrose used for the final 0.2 M sucrose solution, 17.12 g of sucrose is dissolved in 250 mL of MilliQ water. The remaining 1 M sucrose solution that is not used can either be discarded or stored at 4 °C for up to two weeks.

**8. 5 mM umbelliferone stock solution**

**Table:** 

Reagent	Final concentration	Amount or volume
Umbelliferone	5 mM	0.81 mg
DMSO	n/a	1 mL

Due to the volatility of EtOH, it is recommended to dissolve umbelliferone in DMSO, rather than EtOH. The umbelliferone stock solution in DMSO (5 mM) should be stored at -20 °C for long-term storage. It is recommended to aliquot the DMSO stock solutions of umbelliferone prior to storage at -20 °C to minimize freeze-thaw cycles.

**Laboratory supplies**

1. Amber glass vial (Millipore Sigma, catalog number: 27046-U)

2. Black, flat-bottomed 96-well plate (Fisher Scientific, catalog number: M33090)

3. Microcentrifuge tubes (1.5 mL) (Fisher Scientific, catalog number: 05-408-130)

4. Microcentrifuge tubes (0.6 mL) (Fisher Scientific, catalog number: 05-408-120)

5. Motorized tissue grinder (Fisher Scientific, catalog number: 12-141-361)

6. Pellet pestles (Fisher Scientific, catalog number: 749521-1500)

## Equipment

1. Microcentrifuge (Fisher Scientific, catalog number: 75002446, model: Sorvall Legend Micro 21R)

2. Microplate reader for BCA (Agilent Technologies, catalog number: 209924, model: BioTek Synergy 2 SL)

3. Microplate reader for umbelliferone detection (Molecular Devices, model: SpectraMax iD3)

## Procedure

**A. Preparation of tissue lysates**

1. Add pre-chilled detergent-free lysis buffer (10 μL of buffer per mg of tissue) to frozen tissue in a 1.5 mL microcentrifuge tube and homogenize using the preferred method.

*Notes:*

*1. For reference, approximately 30–35 mg of cortex tissue will yield a protein concentration of approximately 10 μg/µL, 40–50 mg of liver tissue will yield a protein concentration of approximately 25 μg/µL, and 10–20 mg of spleen tissue will yield a protein concentration of approximately 12 μg/µL. These tissue amounts are typically enough to detect sufficient enzyme activity (e.g., signal-to-background ratio ≥ 5).*

*2. This protocol was optimized using frozen tissue, but freshly isolated tissue can be used based on the experimenter’s needs.*

*3. While optimizing this protocol, a mini handheld motorized tissue grinder was used; tissues were homogenized for 30 s and then immediately placed on ice until the next centrifugation step. However, this can be adapted per the experimenter’s available resources. For example, a tabletop bead mill homogenizer or probe tip sonicator (e.g., sonicate 10 times total, 1 s on, 1 s off at 20% power; immediately place samples back on ice) could be used instead.*

2. To prevent tissue degradation, immediately place tissue homogenate on ice until all tissue samples are homogenized and ready for centrifugation.

3. Once all samples are homogenized, centrifuge tissue homogenates at 5,000× *g* for 10 min at 4 °C.

**Pause point**: If performing the aCDase activity assay immediately, keep supernatants on ice and use the Pierce BCA Protein Assay kit to determine the protein concentration (µg/µL) of the supernatants. Dilute samples in 0.6 mL microcentrifuge tubes to the desired concentration (e.g., 10 μg per 25 μL of solution added to each well) using cold 0.2 M sucrose solution. If the experimenter intends to perform the aCDase activity assay at a later time point, store supernatants at -80 °C.

*Notes:*

*1. It is not recommended to store samples diluted in 0.2 M sucrose solution for future use. Experimenters should either use freshly diluted samples for the enzyme assay or store the supernatants until ready to prepare fresh sample dilutions and perform the assay.*

*2. It is not recommended to analyze supernatants after multiple freeze-thaw cycles, as this could potentially lead to loss of enzyme activity (e.g., indicated by a lower signal-to-background ratio). To avoid this issue, store single-use sample aliquots at -80 °C for future use.*

**B. Preparation of standard curve**

1. Serially dilute 5 mM umbelliferone stock in 1.5 mL microcentrifuge tubes using DMSO to prepare standard curve stock solutions (**Table 1**).

2. Dilute the stock solutions 200× to generate the final 1× standard curve by combining with 25 mM sodium acetate buffer and 0.2 M sucrose solution (**Table 2**).

*Notes:*

*1. The included recipe (* ***Table 2*** *) makes a total volume of 100 μL and is sufficient for the plating of one well per standard curve point. It is recommended to triple the recipe, e.g., combine 1.5 μL of umbelliferone stock solution, 223.5 μL of 25 mM sodium acetate buffer, and 75 μL of 0.2 M sucrose solution in a 1.5 mL microcentrifuge tube, so each standard curve point can be plated in duplicate, and pipetting errors that could potentially occur with small volumes (e.g., 0.5 μL) can be prevented.*

*2. The range of the 1× standard curve might need to be adapted based on the experimenter’s needs. For example, a standard curve range of 0–200 μM was required for GBA1^+/+^ tissue, but this range might need to be adjusted based on aCDase expression in different animal models and/or tissue types. For this reason, a larger range of 200× standard curve stock is provided to aid in the adaptability of this protocol.*

3. Plate 100 μL of each standard curve point in duplicate into a 96-well plate.

**Table 1.** Preparation of standard curve stock solutions

Tube	Volume and source of umbelliferone (μL)	Volume of DMSO (μL)	Final stock concentration (µM)
A	1,000 of 5 mM stock	0	5,000
B	400 of Tube A	100	4,000
C	250 of Tube B	250	2,000
D	250 of Tube C	250	1,000
E	250 of Tube D	250	500
F*	200 of Tube E	300	200
G*	250 of Tube F	250	100
H*	250 of Tube G	250	50
I*	300 of Tube H	200	30
J*	333.3 of Tube I	166.7	20
K*	250 of Tube J	250	10
L*	250 of Tube K	250	5

*Tubes F–L will be further diluted to prepare the final 1× standard curve.

**Table 2.** Preparation of the final 1× standard curve

Starting stock concentration (µM)	Volume of stock solution (μL)	Volume of 25 mM sodium acetate (μL)	Volume of 0.2 M sucrose (μL)	Final standard concentration (µM)
200	0.5 of Tube F	74.5	25	1
100	0.5 of Tube G	74.5	25	0.500
50	0.5 of Tube H	74.5	25	0.250
30	0.5 of Tube I	74.5	25	0.150
20	0.5 of Tube J	74.5	25	0.100
10	0.5 of Tube K	74.5	25	0.050
5	0.5 of Tube L	74.5	25	0.025
0	0.5 of DMSO	74.5	25	0

**C. Fluorogenic assessment of aCDase activity in tissue lysates**

1. Plate 100 μL of each standard curve point in duplicate to a black flat-bottomed 96-well plate.

2. Plate 25 μL of sample (tissue lysate diluted in 0.2 M sucrose solution) in triplicate.

3. Plate 25 μL of a sample blank in triplicate consisting of 0.2 M sucrose solution without protein.

*Note: As an alternative experimental design, the experimenter can plate a sample blank consisting of protein in 0.2 M sucrose; then, 25 mM sodium acetate buffer without Rbm14–12 substrate can be added to the wells. This could be useful if the experimenter intends to use this protocol to test different tissue types that have higher baseline levels of autofluorescence (e.g., kidney tissue).*

4. Using a multi-channel pipette, add 75 μL of room temperature (20–25 °C) assay solution (e.g., 5 μM Rbm14-12 substrate/25 mM sodium acetate buffer) to each sample and to the sample blank well.

*Notes:*

*1. It is important that the assay solution (Rbm14-12 substrate/25 mM sodium acetate buffer) is only added to the sample and sample blank wells; this solution is not added to the standard curve wells.*

*2. The components in each well are not mixed by pipetting; each solution is added directly to each well without agitation, preventing the generation of bubbles. The total volume of all wells is 100 μL.*

5. Protect the plate from light using aluminum foil and incubate the plate at 37 °C for 3 h without agitation.

6. After 3 h, use a multi-channel pipette to add 50 μL of MeOH, including the standard curve wells.

7. After adding MeOH, immediately add 100 μL of room temperature (20–25 °C) NaIO_4_ solution to all wells, including the standard curve wells.

*Note: The final total volume of all wells is 250 μL.*

8. Protect the plate from light using aluminum foil and incubate the plate at 37 °C for 1 h without agitation.

*Note: The components in each well are not mixed by pipetting; each solution is added directly to each well without agitation, preventing the generation of bubbles.*

**Figure 1.**
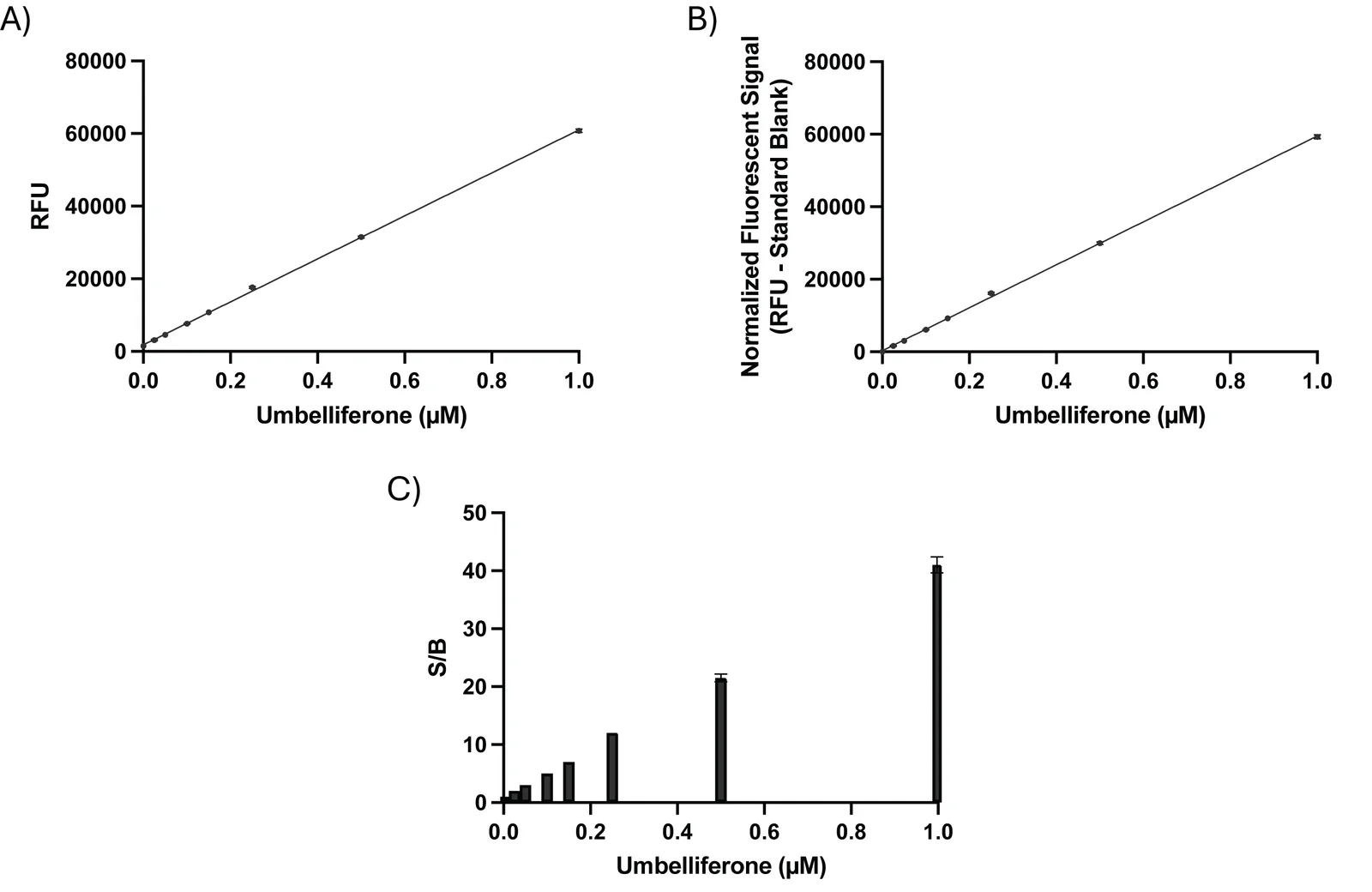
Umbelliferone standard curve. (A) Relative fluorescence units (RFU) (n = 2). The simple linear regression parameters were y-intercept = 1808, m = 59224, and R^2^ = 0.9995. (B) Normalized fluorescence signal, or the difference between RFU and the standard curve blank (a solution without umbelliferone containing the same 25 mM sodium acetate and 0.2 M sucrose solution). The simple linear regression parameters were y-intercept = 321.7, m = 59224, and R^2^ = 0.9995. (C) Signal-to-background (S/B) ratio for each point of the umbelliferone standard curve ranging from 1 to 42 (n = 2). Error bars indicate standard deviation.

9. After 1 h, measure the released fluorescence using a microplate fluorescence reader (excitation: 360 nm; emission: 446 nm).

10. Calculate the amount of released umbelliferone from the fluorescence intensity using the calibration standard curve with umbelliferone (for an example, see **Figure 1**).

## Data analysis

Statistical analyses for the included validation experiments were performed using GraphPad Prism Version 10.5.0. However, the following statistical analyses could easily be performed in other statistical software, such as R, MATLAB, and Python, depending on what is accessible to the experimenter. For enzyme kinetics analyses, simple linear regression was performed for each substrate concentration and its respective substrate incubation period (e.g., 3 h), and the slope was used to represent V_0_. Using V_0_ from a range of substrate concentrations and substrate incubation periods, nonlinear regression (Michaelis–Menten) was performed to yield values for V_max_, K_M_, and R^2^. Catalytic efficiency was calculated as the ratio of V_max_/K_M_ and used as an indicator of how efficiently aCDase hydrolyzes the Rbm14-12 substrate, as previously reported [13]. It is recommended to evaluate each biological sample in triplicate with a minimum of two technical replicates.

## Validation of protocol

**A. Linearity of the fluorogenic assessment with an umbelliferone concentration**

A 74.5 μL volume of 25 mM sodium acetate buffer (pH 4.5), a 0.5 μL volume of umbelliferone standard curve stock solution in DMSO (final DMSO concentration 0.5%), and a 25 μL volume of 0.2 M sucrose solution were added in duplicate to wells of a black, flat-bottomed 96-well plate for each point of the umbelliferone standard curve. The total volume in each well was 100 μL, and the plate was incubated at 37 °C for 3 h, protected from light. After 3 h, 50 μL of MeOH and 100 μL of NaIO_4_ solution (2.5 mg/mL NaIO_4_ in 100 mM glycine/NaOH buffer, pH 10.6) were added to each well, bringing the total volume of each well up to 250 μL. The plate was incubated at 37 °C for an additional hour without agitation, then RFU was detected at excitation 360 nm and emission 446 nm (**Figure 1A**). The normalized fluorescent signal for the umbelliferone standard curve was calculated by taking the difference of RFU of each standard curve point and the standard blank, which consists of 25 mM sodium acetate buffer (pH 4.5), DMSO, and 0.2 M sucrose solution without umbelliferone (**Figure 1B**). The linear regression parameters based on this normalized fluorescent signal were used to calculate the umbelliferone signal in the fluorogenic assessment of aCDase activity in tissue lysate.

**B. Fluorogenic assessment of aCDase activity in brain tissue lysates**

A protocol to evaluate aCDase activity in tissue lysate was developed based on optimized buffers, protein lysate amount, substrate concentration, and incubation times for WT mouse brain lysates (**Table 3**). Tissue samples were homogenized in a detergent-free lysis buffer containing 1 M Tris base at pH 8, 5 M NaCl, 0.5 M EDTA at pH 8, and MilliQ water. After tissue homogenization and centrifugation, the supernatants were collected and used for protein concentration analysis. The supernatants were diluted in 0.2 M sucrose solution to the desired protein concentration (e.g., 10 μg) prior to performing the fluorescence-based assessment of aCDase activity. Once samples were diluted in 0.2 M sucrose solution, they were plated in a black, flat-bottomed 96-well plate and exposed to the Rbm14-12 substrate (e.g., 5 μM) in a sodium acetate solution at an acidic pH (pH 4.5). The enzymatic reaction (aCDase hydrolysis of the amide bond of Rbm14-12, yielding the corresponding aminodiol) was then quenched by the addition of MeOH. Additionally, NaIO_4_ in glycine/NaOH buffer (pH 10.6) was added to oxidize the reaction intermediate (aminodiol) to form the corresponding oxidized product (aldehyde), which then undergoes β-elimination at basic pH, leading to the formation of umbelliferone (fluorophore generation).

**Figure 2.**
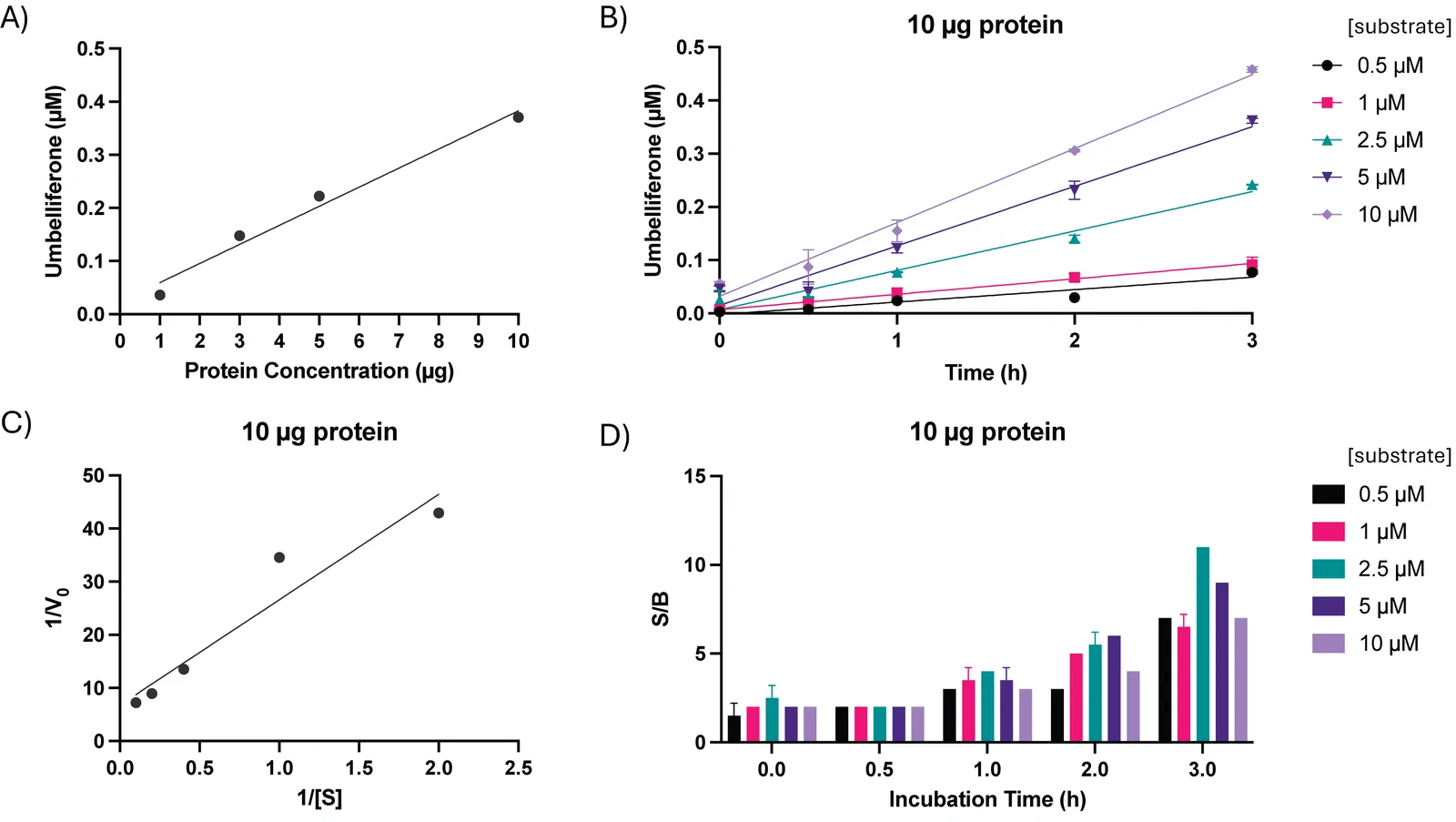
Optimization of the fluorescence-based protocol for measuring acid ceramidase activity in tissue lysate. (A) Assay linearity with varying protein lysate concentration. The fixed substrate concentration was 5 μM, and the substrate incubation period was 3 h. (B) Reaction progress curves of 10 μg of protein incubated in the presence of varying substrate concentrations for the indicated time periods (n = 2). A simple linear regression was performed for each substrate concentration, and the slope was used to represent V_0_. (C) Lineweaver–Burk plot of Rbm14-12 hydrolysis with 10 μg of protein and a 3-h substrate incubation period. The nonlinear regression (Michaelis–Menten) parameters were V_max _= 0.2058 μM/h, K_M_ = 4.590 μM, and R^2^ = 0.9903. (D) Signal-to-background (S/B) ratio of Rbm14-12 hydrolysis with 10 μg of protein at varying substrate concentrations and substrate incubation periods (n = 2). Error bars indicate standard deviation.

During a 3-h substrate incubation period, hydrolysis of the Rbm14-12 substrate at a fixed substrate concentration of 5 μM showed a linear response from 1 to 10 μg of protein derived from WT brain tissue lysate (**Figure 2A**). Kinetic studies evaluating substrate incubation periods ranging from 0 to 3 h and using 10 μg of protein derived from WT mouse brain tissue lysates yielded a maximal catalytic velocity of aCDase (V_max_) of 0.2058 μM/h and a Michaelis–Menten constant (K_M_) of 4.590 μM (**Figure 2B and C**). Using 3 and 5 μg of protein yielded comparable K_M_ values: 4.206 μM and 4.213 μM, respectively (**Figure S1**). The V_max_ of aCDase using 10 μg of protein was higher compared to using lower protein lysate concentrations, such as 3 μg (V_max_ = 0.0626 μM/h) and 5 μg (V_max_ = 0.0989 μM/h) (**Figure S1**). Because V_max_ was higher using 10 μg of protein, catalytic efficiency (V_max_/K_M_) of aCDase using 10 μg of protein (V_max_/K_M_ = 0.045 h^-1^) was also higher compared to using lower protein lysate concentrations, such as 3 μg (V_max_/K_M_ = 0.015 h^-1^) and 5 μg (V_max_/K_M_ = 0.023 h^-1^), indicating that aCDase is more efficient in converting the Rbm14-12 substrate under the 10 μg condition (**Figure S1**). With respect to substrate concentrations ranging from 0.5 to 10 μM and a 3-h substrate incubation, the substrate concentration that yielded a [substrate]/K_M_ ratio closest to the value of 1 was 5 μM (**Figure S3**).

Kinetic studies evaluating substrate incubation periods ranging from 0 to 4 h and using 10 μg of protein derived from WT mouse brain tissue lysates yielded a V_max_ of 0.2166 μM/h and a K_M_ of 4.079 μM (**Figure S2**). Using 3 and 5 μg of protein yielded lower K_M_ values: 2.436 μM and 2.935 μM, respectively (**Figure S2**). Using substrate incubation periods ranging from 0 to 4 h, the V_max_ of aCDase using 10 μg of protein remained higher compared to using lower protein lysate concentrations, such as 3 μg (V_max _= 0.0563 μM/h) and 5 μg (V_max _= 0.0987 μM/h) (**Figure S1**). During a longer substrate incubation period of up to 4 h, the V_max_/K_M_ of aCDase using 10 μg of protein (V_max_/K_M_ = 0.053 h^-1^) also remained higher compared to using lower protein lysate concentrations, such as 3 μg (V_max_/K_M_ = 0.023 h^-1^) and 5 μg (V_max_/K_M_ = 0.034 h^-1^), suggesting higher catalytic efficiency under the 10 μg condition (**Figure S2**). With respect to substrate concentrations ranging from 0.5 to 10 μM and a 4-h substrate incubation, the substrate concentration that yielded a [substrate]/K_M_ ratio closest to the value of 1 was 5 μM (**Figure S3**).

**Table 3.** Fluorescence-based protocol for measuring acid ceramidase activity in tissue lysate

Step	Action
1	Homogenize tissue in detergent-free lysis buffer, then centrifuge samples and collect supernatant.
2	Determine protein concentration of collected supernatants (samples).
3	Dilute samples in 0.2 M sucrose solution to the final desired protein lysate concentration and plate in triplicate in a 96-well plate (25 μL/well).
4*	Plate sample blank (0.2 M sucrose without protein) in triplicate in a 96-well plate (25 μL/well).
5	Add assay solution (25 mM sodium acetate and Rbm14-12 reconstituted in DMSO) to each sample and sample blank well (75 μL/well).
6	Wrap the 96-well plate in foil to protect from light and incubate at 37 °C for 3 h.
7	After 3 h of substrate incubation, add 50 μL of MeOH, immediately followed by 100 μL of NaIO_4_ solution.
8	Wrap the 96-well plate in foil to protect from light and incubate at 37 °C for 1 h.
9	After 1 h, quantify released fluorescence using a microplate fluorescence reader (excitation: 360 nm; emission: 446 nm).
10	Calculate the amount of released umbelliferone from the fluorescence intensity using a standard curve.

*See General note 5 for the use of an aCDase inhibitor.

**C. Discussion**

We present a protocol to measure aCDase activity in tissue lysates using the fluorogenic Rbm14-12 substrate, which resembles the naturally occurring Cer and is hydrolyzed by aCDase at acidic pH, as previously reported [13]. Upon enzymatic amide cleavage of Rbm14-12, an aminodiol is generated, which undergoes oxidation in the presence of NaIO_4_ [13,25]. The resulting oxidized product then undergoes β-elimination at basic pH mediated by a glycine/NaOH buffer. This reaction generates umbelliferone, which absorbs UV light and emits fluorescence that can be read on a microplate reader at Ex/Em = 360/446 nm [13,25]. To our knowledge, this is the first thoroughly described protocol for the measurement of aCDase activity in tissue, as demonstrated using WT mouse brain tissue. A previous study performed the ex vivo measurement of aCDase activity in liver tissue [23]. Other methods to measure aCDase activity in different rodent tissues use LC/MS-based or radiometric methodologies [26–30]; however, many institutions are moving away from radioactivity, and LC/MS methods are more laborious and may not always be available. Before the discovery of Rbm14-12 as a specific aCDase substrate, other coumarinic substrates were used in a fluorescence-based assay using lysosomal and microsomal extracts from rat liver as enzyme source [31]. All other recent work related to the measurement of aCDase activity has been limited to cell pellet lysates or intact cell-based studies using radiometric methods, LC/MS, fluorescence-based methodologies, thin layer chromatography, or conjugated Cer [13,16,18,26,28,32–41].

When evaluating aCDase activity in cell pellets, activity levels are typically on the picomolar scale [13,42,43]; however, during the optimization process using brain lysate, we determined that aCDase activity is consistently higher, necessitating the use of a micromolar scale. Additionally, for cell-based analyses, cell pellets are often sonicated in a 0.2 M sucrose solution and used as protein extracts for the assay [13,34,42,43]. However, for tissue-based analyses, we determined it was best to homogenize tissue samples in a solution containing 1 M Tris, 5 M NaCl, 0.5 M EDTA, and MilliQ water, followed by centrifugation. It is critical that no detergents are present in any of the assay solutions, especially the lysis buffer. In our own troubleshooting, we could not detect the release of umbelliferone fluorescence when Triton X-100 was included in the lysis buffer. It has been suggested that the micelles in detergents engulf the Rbm14-12 substrate and inhibit hydrolysis by preventing the substrate from interacting with the catalytic Cys143 residue of aCDase [13]. For all previous cell-based analyses utilizing an Rbm substrate, EtOH has typically been used as the organic solvent for the Rbm substrate and the umbelliferone standard curve [13,34,42,43]. We observed that DMSO was a better alternative for a couple of reasons: (i) DMSO is less volatile than EtOH and allows for the Rbm substrate to be stably stored at -20 °C for a longer amount of time; and (ii) when DMSO is used in the umbelliferone standard curve, the simple linear regression parameters of the curve remains consistent over time; when EtOH is used, the simple linear regression parameters change over time during the different incubation cycles in the assay due to solvent evaporation.

Using WT mouse brain tissue, 10 μg of protein and 5 μM substrate were determined as the ideal protein lysate amounts and substrate concentrations for this assay; however, this could differ based on animal model, tissue region, and tissue type used for analysis. Not only did the reaction remain linear using 10 μg of protein lysate, but when compared to lower protein lysate amounts, 10 μg of protein yielded higher values for V_max_ and V_max_/K_M_. This indicates that aCDase is more efficient in converting the Rbm14-12 substrate under the 10 μg condition. Using 10 μg of protein also yielded S/B ratios ≥ 5, ensuring the experimenter can distinguish between the umbelliferone signal and background noise [44,45]. Additionally, when compared to lower protein lysate amounts, 10 μg of protein was the only protein lysate amount that yielded a goodness-of-fit (R^2^) ≥ 0.99 for the Michaelis–Menten curves (**Figure S1** and **S2**). It is likely that protein lysate amounts >10 μg would also yield R^2^ values ≥ 0.99, but these data suggest that 10 µg protein is sufficient, allowing the experimenter to conserve the amount of tissue lysate consumed in the assay. Nonetheless, it should be noted that this desired protein lysate amount could differ between animal models and/or tissue types.

Using WT mouse brain tissue, 5 μM substrate was determined as the ideal substrate concentration for this assay, although this could differ based on animal model, tissue type, and tissue region used for analysis. As previously mentioned, the hydrolysis of the Rbm14-12 substrate at 5 μM is linear at the desired protein lysate amount of 10 μg (**Figure 2A**). Additionally, kinetics analysis using WT mouse brain tissue, 10 μg of protein, and up to 3-h substrate incubation yielded a K_M_ of 4.59 μM (**Figure 2C**). The [substrate]/K_M_ ratio using 5 μM substrate was equivalent to 1.09, suggesting 5 μM as the optimal substrate concentration, along with a 3-h substrate incubation period (**Figure S3**). The S/B ratios of 10 μg of protein in the presence of varying substrate concentrations varied, with 2.5 μM substrate and a 3-h substrate incubation yielding the highest S/B (**Figure 2D**). However, based on the [substrate]/K_M_ ratio using 2.5 μM substrate ([substrate]/K_M_ = 0.545), 2.5 μM substrate concentration is <1 (**Figure S3**). Using a 2.5 μM substrate concentration would be sub-optimal, because when [substrate]/K_M_ = 1, this indicates that aCDase is operating at 50% capacity. When [substrate]/K_M_ = 1, subtle or overt changes in enzyme performance can be detected without having to worry about enzyme saturation or the lack of enzyme present [46]. The S/B ratio starts to decline when 10 μg of protein is in the presence of 10 μM substrate (**Figure 2D**). The S/B ratio is second-highest when 10 μg of protein is in the presence of 5 μM substrate during a 3-h substrate incubation (**Figure 2D**). Thus, based on the [substrate]/K_M_ ratio and S/B ratio, 5 μM substrate is the optimal substrate concentration with a 3-h substrate incubation period. However, since the difference between using 10 μg of protein and a 3-h substrate incubation versus a 4-h substrate incubation was marginal, it should be noted that a substrate incubation time longer than 3 h may be required if a tissue type with low aCDase expression is being tested. This can be modified based on the experimenter’s conditions.

This assay is efficient, with the time required to prepare the buffers, prepare the samples, and run the assay being approximately 6 h. It is specific for the forward reaction of aCDase (e.g., Cer hydrolysis) due to the acidic pH and Rbm14-12 substrate used in the assay. Furthermore, the only equipment needed for this assay is a tissue homogenizer, centrifuge, incubator, and a microplate fluorescence reader, making it feasible to conduct this assay in most laboratory settings. This versatile procedure is compatible with both central (e.g., brain) and peripheral (e.g., liver, lungs, and spleen) tissues, with the potential to be optimized for various tissue types based on the experimenter’s needs. This assay could help researchers easily validate FD and SMA-PME animal models by characterizing the extent of aCDase activity deficiency in different tissue types. Furthermore, it could help researchers study the efficacy and target engagement of aCDase-based therapeutics, which is becoming increasingly relevant in oncology and neurodegenerative disease, thereby expanding the utility of this assay beyond a single research field.

## General notes and troubleshooting

**General notes**

1. For mouse tissue sample collection, it is recommended to transcardially perfuse the animal with 0.9% saline (with 10 U/mL heparin and 0.5% w/v sodium nitroprusside) at 9 mL/min for a total of 25 mL [48]. After tissue dissection, immediately snap-freeze the tissue and store at -80 °C until ready to prepare the tissue homogenate for the aCDase activity assay.

2. It is recommended not to use detergents, such as Triton X-100, sodium cholate, or CHAPS in any of the solutions, especially the lysis buffer, as previously reported using cell lysates, to avoid interference in the umbelliferone signal [13].

3. Since the purpose of this protocol is to assess the activity of lysosomal aCDase, the pH of this assay is maintained acidic using sodium acetate buffer at pH 4.5.

4. The umbelliferone standard curve is prepared using sodium acetate buffer, umbelliferone dissolved in DMSO, and 0.2 M sucrose solution. The assay samples are prepared using sodium acetate buffer, Rbm14-12 dissolved in DMSO, and protein lysate diluted in 0.2 M sucrose solution. The sample blank is prepared using the same solution as the assay samples, except without the protein extracts. It is important that these components remain consistent to ensure that the standards and the samples are being tested under the same conditions.

5. As previously reported using a cell lysate-based assay, the sample blank can be used as the negative control sample [13]. Alternatively, the experimenter can prepare a separate assay solution containing the Rbm14-12 substrate, sodium acetate buffer, 0.2 M sucrose solution, and an aCDase inhibitor, such as carmofur (MedChemExpress, catalog number: HY-B0182) [26] or acid ceramidase-IN-1 (MedChemExpress, catalog number: HY-141866) [34], and adjust the 96-well plate design accordingly to include samples treated with Rbm14-12 in triplicate and samples treated with Rbm14-12 plus an aCDase inhibitor in triplicate.

6. A Sorvall Legend Micro 21R microcentrifuge was used to centrifuge tissue homogenates, but any centrifuge can be used if it can be set to 4 °C and 5,000× *g*.

7. A Biotek Synergy 2 SL microplate reader was used for the Pierce BCA Protein Assay, but any microplate reader can be used if it can detect absorbance at 562 nm.

8. A SpectraMax iD3 microplate reader was used to detect umbelliferone fluorescence, but any microplate reader can be used if it can have the following settings (excitation/emission): 360 nm/446 nm.

9. This protocol was optimized using frozen tissue samples, but freshly isolated tissue could be used.

**Troubleshooting**

**Problem 1**: Challenges related to starting the optimization process.

Possible cause: Not knowing which parameters to optimize first and how.

Solution: It is recommended to start the optimization process by determining the optimal protein lysate amount and substrate concentration at different incubation times. Use a range of substrate incubation times (e.g., 0–4 h), a range of protein lysate concentrations (e.g., 1, 5, 10, 25, 50 μg), and a range of substrate concentrations (e.g., 0.5, 1, 2.5, 5, 10 μM). It is recommended that each substrate's incubation time be evaluated in separate 96-well plates. For example, if you were to assess Rbm14-12 hydrolysis after 0, 0.5, 1, 2, 3, and 4 h of substrate incubation, six separate 96-well plates would be needed, and within each individual 96-well plate, the same range of protein lysate concentrations and substrate concentrations could be assessed. With this initial set of analysis, the experimenter can assess reaction linearity, signal-to-background (S/B) ratios, V_max_, K_M_, V_max_/K_M_, and [substrate]/K_M_. Based on these preliminary results and key considerations outlined below, the experimenter can modify the protein lysate concentration and substrate ranges, if needed, and then repeat the analyses to find the optimal assay parameters. As outlined below, the experimenter should choose assay parameters that yield a linear reaction, S/B ratios ≥ 5, [substrate]/K_M_ close to the value of 1, and high catalytic efficiency.

**Problem 2**: Optimizing protocol parameters to yield signal-to-background (S/B) ratios ≥ 5.

Possible cause: aCDase expression can differ based on tissue type and potentially by region within the same tissue.

Solutions:

a. Samples: aCDase expression can differ based on tissue type and potentially by region within the same tissue. Optimize experimental conditions (e.g., protein lysate concentration or substrate concentration) to yield sample S/B ratios ≥ 5. In other words, the mean signal of your sample (the average RFU) of your sample divided by the mean signal of your sample blank (the average RFU of your sample blank) should be ≥5. Having a sample’s S/B ratio ≥ 5 ensures the umbelliferone signal is distinguishable from background noise, which is why this is an important parameter to measure during optimization (Figure 1) [44,45].

b. Standard curve: Confirm that the experimental S/B ratios are within the dynamic range of the standard curve S/B ratios. For example, if your samples have S/B ratios between 5 and 20, the range of S/B ratios in the standard curve needs to include these values. The optimized standard curve concentration range in this protocol is from 0 to 1 μM with an S/B ratio range of 1–42 (**Figure 1C**), because the experimental S/B ratios consistently fell within this range (**Figure 2D**). This concentration and S/B ratio range can be altered and optimized based on the experimenter’s needs. However, it is important that the S/B ratio of the samples always falls within the S/B ratio range of the standard curve, as demonstrated previously (**Figure 1C** and **Figure 2D**), because this guarantees that the readout of the samples is within the detection range of the standard curve and can be quantified reliably [44,45].

**Problem 3**: Optimizing protein lysate amount.

Possible cause: aCDase expression can differ based on tissue type and potentially by region within the same tissue.

Solution: Protein lysate amount needs to be optimized based on tissue type and region. It is recommended to first test a wide range of protein lysate amounts (e.g., 1, 10, 50, 100 μg) at a fixed substrate concentration to assess linearity. This assessment will show which protein lysate concentrations yield a linear reaction. Protein lysate concentrations yielding nonlinear results should not be used. Based on these results, reduce the linearity assessment to a smaller protein concentration range (e.g., 1, 3, 5, 10 μg) at a fixed substrate concentration. This assessment will allow the experimenter to identify the optimal amount of protein required to detect the umbelliferone signal within a linear range.

Along with this linearity assessment, evaluate the kinetics of Rbm14-12 hydrolysis using a range of protein lysate concentrations, substrate concentrations, and substrate incubation periods (e.g., 0–3 h) to determine which protein lysate concentration yields higher catalytic efficiency (V_max_/K_M_). For example, after determining an ideal protein lysate amount range of 1–10 μg and deciding that 10 μg yields the best S/B ratios (**Figure S4**), evaluate this protein lysate concentration range exposed to a range of Rbm14-12 substrate concentrations (e.g., 0.5, 1, 2.5, 5, and 10 μM) for 0, 0.5, 1, 2, and 3 h to determine which conditions yield higher catalytic efficiency of Rbm14-12 hydrolysis (**Figure S1**).

**Problem 4**: Optimizing substrate concentration.

Possible cause: aCDase expression can differ based on tissue type and potentially by region within the same tissue.

Solution: Rbm14-12 substrate concentration needs to be optimized based on tissue type and region to ensure linearity of reaction and S/B ratios ≥ 5. It is recommended to test a wide range of substrate concentrations (e.g., 1, 5, 10, 50, 100 μM) in combination with ranging protein lysate concentrations. Based on these results, check linearity within a smaller μM range (e.g., 1, 2.5, 5, 10, 20, 25 μM). Along with linearity assessment, evaluate the kinetics of Rbm14-12 hydrolysis using a range of protein lysate concentrations, substrate concentrations, and substrate incubation periods to determine which substrate concentration yields a [substrate]/Km ratio closest to the value of 1, to ensure high sensitivity of the assay (**Figure S3**) [46].

**Problem 5**: Optimizing substrate incubation period.

Possible cause: The length of the substrate incubation period can affect reaction linearity.

Solution: When choosing the optimal Rbm14-12 substrate incubation time period, the experimenter should assess reaction linearity, the amount of protein needed, fluorescence intensity, S/B ratios, and kinetics of substrate hydrolysis. If the substrate incubation period is too long, the reaction can become nonlinear. If the substrate incubation is too short, the fluorescence intensity could be too low (e.g., S/B ratios could be <5 and indistinguishable from background noise). The substrate incubation period could also impact V_max_/K_M_ and [substrate]/K_M_ ratios, so these factors must be considered for optimal sensitivity.

## Supplementary information

The following supporting information can be downloaded here:

1. **Dataset S1**. Data corresponding to Figure 1

2. **Dataset S2**. Data corresponding to Figure 2

3. **Dataset S3**. Kinetics analysis of Rbm14-12 hydrolysis corresponding to **Figure S1**

4. **Figure S1**. Kinetics analysis of Rbm14-12 hydrolysis using 0, 0.5, 1, 2, and 3 h substrate incubation periods

5. **Dataset S4**. Kinetics analysis of Rbm14-12 hydrolysis corresponding to **Figure S2**

6. **Figure S2**. Kinetics analysis of Rbm14-12 hydrolysis using 0, 0.5, 1, 2, 3, and 4 h substrate incubation periods

7. **Figure S3**. [substrate]/K_M_ ratios for 3- vs. 4-h substrate incubation periods

8. **Dataset S5**. S/B ratio data corresponding to Figure S4

9. **Figure S4**. A comparison of S/B ratios for 3, 5, and 10 μg of protein exposed to a range of Rbm14-12 substrate concentrations and substrate incubation periods

10. **Template 1**. aCDase activity assay reagent calculator
